# Polycomb Mediated Epigenetic Silencing and Replication Timing at the *INK4a/ARF* Locus during Senescence

**DOI:** 10.1371/journal.pone.0005622

**Published:** 2009-05-20

**Authors:** Hanane Agherbi, Anne Gaussmann-Wenger, Christophe Verthuy, Lionel Chasson, Manuel Serrano, Malek Djabali

**Affiliations:** 1 Centre d'immunologie INSERM-CNRS de Marseille Luminy, Marseille, France; 2 Spanish National Cancer Research Center (CNIO), Madrid, Spain; Roswell Park Cancer Institute, United States of America

## Abstract

**Background:**

The *INK4/ARF* locus encodes three tumor suppressor genes (p15^Ink4b^, Arf and p16^Ink4a^) and is frequently inactivated in a large number of human cancers. Mechanisms regulating *INK4/ARF* expression are not fully characterized.

**Principal Findings:**

Here we show that in young proliferating embryonic fibroblasts (MEFs) the Polycomb Repressive Complex 2 (PRC2) member EZH2 together with PRC1 members BMI1 and M33 are strongly expressed and localized at the *INK4/ARF* regulatory domain (RD) identified as a DNA replication origin. When cells enter senescence the binding to RD of both PRC1 and PRC2 complexes is lost leading to a decreased level of histone H3K27 trimethylation (H3K27me3). This loss is accompanied with an increased expression of the histone demethylase Jmjd3 and with the recruitment of the MLL1 protein, and correlates with the expression of the Ink4a/Arf genes. Moreover, we show that the Polycomb protein BMI1 interacts with CDC6, an essential regulator of DNA replication in eukaryotic cells. Finally, we demonstrate that Polycomb proteins and associated epigenetic marks are crucial for the control of the replication timing of the *INK4a/ARF* locus during senescence.

**Conclusions:**

We identified the replication licencing factor CDC6 as a new partner of the Polycomb group member BMI1. Our results suggest that in young cells Polycomb proteins are recruited to the *INK4/ARF* locus through CDC6 and the resulting silent locus is replicated during late S-phase. Upon senescence, Jmjd3 is overexpressed and the MLL1 protein is recruited to the locus provoking the dissociation of Polycomb from the *INK4/ARF* locus, its transcriptional activation and its replication during early S-phase. Together, these results provide a unified model that integrates replication, transcription and epigenetics at the *INK4/ARF* locus.

## Introduction

Cellular senescence is a fundamental cellular program that is activated after a finite number of cell divisions and operates to avoid further cell proliferation. In addition cellular senescence constitutes a tumor suppressor mechanism [1], [2]. The tumor suppressor pathways, ARF/MDM2/p53 and p16^INK4a^/Rb, have been shown to play critical roles in the induction of cellular senescence [3]. Studies on Polycomb group genes (Pc-G) have demonstrated that beside their function in controlling the expression of Hox genes, Pc-G play a central role in cell proliferation through the repression of the *INK4/ARF* locus [4], [5], [6]. This locus encodes both p16^INK4a^, which prevents inactivation of the tumor suppressor RB, and p19^ARF^, which stabilizes the tumor suppressor p53 [7], [8]. Evidence supporting the direct control of the cell cycle by Pc-G proteins in vertebrates came from studies on mouse *Bmi1* mutants. Bmi1 was first identified as a proto-oncogene that cooperates with c-Myc to promote the generation of mouse B- and T-lymphomas [9]. Mice lacking *Bmi1* exhibit strong proliferative defects during lymphocyte development. In the absence of Bmi1, M33, or Phc2, primary embryonic fibroblasts (MEFs) are unable to progress into S phase, undergo premature senescence after only a few passages in culture and show an increased accumulation of the tumor suppressors p16^INK4a^, p19^ARF^ and p15^INK4b^ [4], [10]. Generation of *Bmi1/Ink4a/Arf* compound mutant mice have provided genetic evidence that at least part of these defects are due to activation of the *INK4a/ARF* locus [11].

Pc-G and Trx-G proteins function in distinct multiprotein complexes which control transcription by altering the structure of chromatin, organizing it into either a “closed” or an “open” conformation. The Trx-G protein MLL1 mediates lysine-directed histone methylation [12], [13]. Methylation on lysine-4 of histone H3 (H3K4me2 and H3K4me3) is associated with a permissive and transcriptionally active state of the chromatin [14]. Pc-G proteins are transcriptional repressors that functionally can be separated into at least two different complexes: the initiation complex, Polycomb complex 2 (PRC2), which in humans consists of EZH2, EED, and SUZ12; and the maintenance complex, PRC1, with the core proteins RNF2, HPC, and BMI1. Both PRC1 and PRC2 complex members have been linked to cell cycle control. EZH2 is the active component of PRC2 through its SET domain histone methyltransferase activity specific for Lys 27 (K27) of histone H3 and K26 of histone H1 [15], [16]. It has been demonstrated that PRC2 is required for PRC1 binding to chromatin [17], presumably achieved through binding of HPC protein to H3K27me3 [15]. It has recently been shown that Polycomb proteins are bound to the *INK4a/ARF* locus and dissociated during senescence [18]. We have now extended this study and demonstrate that both Polycomb and the trithorax (Trx-G) member MLL1 are localized at this locus and importantly associated to the Regulatory Domain (RD) of the *INK4/ARF* locus identified as a DNA replication origin and as a global transcriptional regulator of the entire locus [19], [20]. Also, we demonstrate that BMI1 interacts with the licencing factor CDC6. Finally, we show that the late timing of replication of the *INK4a/ARF* locus in young proliferating MEFs shifts to an early replication timing in senescent and notably in PRC2 mutant MEFs. Together our results demonstrate that MLL1 and Polycomb group genes directly control the *INK4a/ARF* locus through epigenetic chromatin modifications and that the loss of repressive epigenetic marks both in senescent and Polycomb mutant cells leads to a shift of the replication timing of the *INK4a/ARF* locus.

## Materials and Methods

### Mouse embryonic fibroblasts (MEFs)

Day-12p.c. wild type, *M33* mutant and *Bmi1* mutant embryos were mechanically dissociated into single cells and cultured in DMEM with 10% FCS and penicillin and streptomycin (Invitrogen, Breda, The Netherlands). MEFs were passaged every two to three days and viable cells were counted by Trypan Blue (Invitrogen) exclusion. Senescent cells were detected using the beta-Galactosidase Staining Kit (BioVision) following the manufacturer's recommendations.

### Quantification of mRNA levels by Q-PCR

For reverse transcriptase Q-PCR (Q-RT-PCR) analysis, total RNA was extracted from MEFs with the RNeasy Mini Kit (QIAGEN) according to the manufacturer's protocol. cDNA was synthesized from 1 µg of total RNA using the QuantiTect Reverse Transcription Kit (QIAGEN). Q-PCR amplification of either mouse *Ink4a* transcripts or *Arf* was performed with the SYBR Green PCR Master Mix Kit (Applied Biosystems) in a 7500 Real-Time PCR System From Applied Biosystems. Primers used are given in Table 1. Q-RT-PCR analysis of Gapdh mRNA expression was performed as a positive control and for normalization.

**Table 1:** Sequences of Primers used for ChIP and gene expression analysis

Primer	Sequence 5′-3′
RD^INK4a/ARF^ fwd	TTCCTATTTCGCTGTAGCAAC
RD^INK4a/ARF^ rev	AACTAACCAGGCCTCCTCCCA
Exon 1b fwd	AGGTGCCTCAACGCCGAAG
Exon 1b rev	CTGGTCCAGGATTCCGGTGCGG
Exon 2 fwd	ATGGGCAACGTTCACGTAGCAGC
Exon 2 rev	AGCGGTACACAAAGACCACCCA
p19^ARF^ fwd	TCGCAGGTTCTTGGTCACTGT
p19^ARF^ rev	TGCTACGTGA ACGTTGCCCA
p16^INK4a^ fwd	GTGTGCATGACGTGCGGG
p16^INK4a^ rev	GCAGTTCGAATCTGCACCGTAG
Ezh2 fwd	AGGATACAGCCTGTGCACATCATGA
Ezh2 rev	GATCCAGAACTTCATCCCCCATAT
Bmi-1 fwd	CCAGCAAGTATTGTCCTATTTGTGA
Bmi-1 rev	TTGAAGAGTTTTATCTGACCCTTATGTT
UTX fwd	GCAAGTGCAGATACATGGTGTTC
UTX rev	GCCTAGATCCATCCAGGCTGC
JMJD3 fwd	GGAAGCCACAGCTACAGGAGC
JMJD3 rev	CCCAATAGTGCTCATGTACCGC

### Retroviral transduction of embryonic fibroblasts (MEFs)

Ecotropic virus producer cells (Phoenix) were transfected with pMSCV-Myc-cdc6 or the empty vector (mock) using Lipofectamine™ 2000 Reagent (Invitrogen). Retroviral supernatant was collected after 24 h. For transduction wild type and Bmi^−/−^ MEFs were plated in 6-well plates. Cells were incubated with the viral supernatant and Polybrene (4 µg/ml) for 30 min before centrifugation (2000 rpm, 32°C, 60 min). After 12 h incubation (10% CO_2_, 37°C) cells were washed and incubated with fresh medium.

### Immunocytochemistry

MEFs (wild type and BMI^−/−^) were transduced with pMSCV-Myc-cdc6 or the empty vector (mock) at passage P3. 72 h after transduction, cells were plated on glass coverslips, fixed in 4% paraformaldehyde and permeabilized with cold PBS containing 0.2% Triton X-100. After blocking, the cells were incubated with primary antibodies (mouse anti-Cdc6 (sc-9964; Santa Cruz Biotechnology), rabbit anti-p16^INK4a^ (sc-1207; Santa Cruz Biotechnology), washed with secondary antibodies, stained with DAPI, and mounted before viewing.

### Chromatin immunoprecipitation assays

ChIP were performed and analyzed essentially as described [16]. The antibodies used were rabbit anti-H3acetyl (06-599, Upstate Biotechnology), rabbit anti-H3K27me3 (07-449; Upstate Biotechnology), rabbit anti-M33 (kind gift of Dr. Higashinakagawa, T) [21], rabbit anti-H3K4Me3 (05-745; Upstate Biotechnology), rabbit anti-EZH2 (07-689; Upstate Biotechnology), goat anti-BMI1 (sc-8906; Santa Cruz Biotechnology), anti-MLL (05-765; Upstate Biotechnology). For normal ChIP, the immunoprecipitated DNA was quantified by real-time Q-PCR (Applied Biosystems) and normalized with the 1/5 input. The sequences of the PCR primers are listed in Table 1.

### Coimmunoprecipitations

NIH3T3 cells were transiently transfected with pCG-HAcdc6 using Lipofectamine™ 2000 Reagent (Invitrogen) following the manufacturer's instructions. 72 h after transfection CoIP were performed using Nuclear Complex Co-IP Kit (active motif) with mouse anti-HA (2367; Cell Signaling). Thymocytes were isolated from 5 to 7 weeks old mice and protein extraction and CoIP were performed using Nuclear Complex Co-IP Kit (active motif) with mouse anti-BMI1 (05-637; Upstate Biotechnology). Western blotting procedure and reagents for western blot analysis were previously described [4]. Antibodies used were mouse anti-CDC6 (sc-9964; Santa Cruz Biotechnology), goat anti-BMI1 (sc-8906; Santa Cruz Biotechnology).

### Replication timing analysis

BrdU labeling, fixation in cold 70% ethanol, cell cycle fractionation by flow cytometry and isolation of BrdU-labeled DNA by immunoprecipitation were carried out as previously described [22].

## Results and Discussion

### Polycomb expression and cellular senescence in mouse embryonic fibroblasts

**Figure 1:**
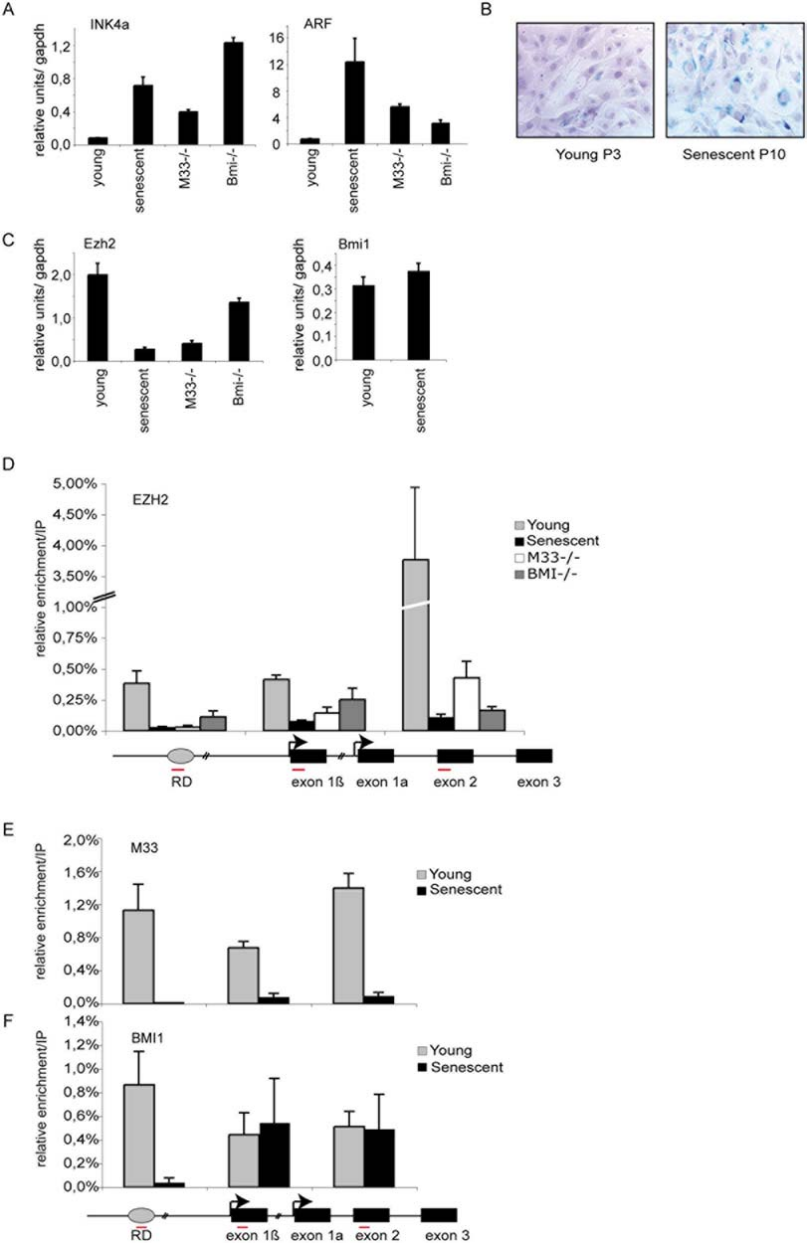
Analysis of Polycomb EZH2, M33 and BMI1 binding at the *INK4a/ARF* region. A) qPCR analysis of p16^INK4a^ and p19^ARF^ in young (P3), senescent (P10) and in *Bmi1* and *M33* mutant MEFs. B) B-galactosidase staining to detect senescent cells at passage 3 (P3) and passage 10 (P10). C) qPCR analysis of the mRNA levels of Polycomb EZH2 and Bmi1 in the indicated cells. D–F) Schematic diagram of the *INK4a/ARF* locus: amplified regions that were tested in ChIP experiments are indicated by red bars (sequences are given in Table 1). Wild type P3 (young), P10–12 (senescent), M33−/− (P4) and Bmi−/− (P4) MEFs were subjected to ChIP assays using anti EZH2, BMI1 and M33 antibodies. DNA enrichment was calculated as described in Materials and Methods. Bars represent the mean+/−s.d. of quantifications from two to four separate immunoprecipitations analyzed in triplicate.

To study cellular senescence we have used serial passaging of mouse embryonic fibroblasts (MEFs) cultured from 12-day-old C57BL/6 embryos. As already described, MEFs were able to undergo a limited number of population doublings (P12) before they senesced. Senescence was assessed by monitoring the endogenous B-galactosidase (B-gal) activity at pH 6 (Fig. 1B). At this stage (P10), cells overexpress both p16^Ink4a^ and p19^Arf^. As expected knock out cells derived from PRC1 members Bmi1 and M33 overexpress both p16^Ink4a^ and p19^Arf^ as soon as passage P3 [4] (Fig. 1A). Since EZH2 is required for Polycomb silencing we measured differences in expression levels of Ezh2 in young and senescent MEFs by Q-RT-PCR (Fig. 1C). Protein levels of EZH2 (not shown) in MEFs correlated with RNA levels; Ezh2 is more abundant in early passage (P3) than in senescent MEFs (P10) (Fig. 1C). In PRC1 mutant cells (M33, Bmi1) the expression level of Ezh2 is strongly diminished. Interestingly, expression level of the PRC1 member Bmi1 is not modified in senescent cells as compared to young proliferating cells (Fig. 1C) [18].

### Polycomb proteins are localized at the RD*^INK4a/ARF^*

The **RD** ***^INK4a/ARF^***has been identified as a putative DNA replication origin that assembles a multiprotein complex containing CDC6 and that coincides with a conserved non coding DNA element identified as a transcriptional regulatory element [20]. Whether PRC1 and PRC2 members bind directly to **RD** ***^INK4/ARF^*** had not been tested yet. In order to assess this question, we performed ChIP assays to examine whether EZH2 directly binds this transcriptional regulatory element. Oligonucleotide primers were designed in order to examine in young proliferating MEFs the distribution of EZH2 at the RD element and along the *INK4a/ARF* locus. We found that EZH2 is bound to the first exon of *ARF*, exon 1b, and with a maximum peak to the shared exon of *INK4a* and *Arf*, exon 2, in early-passage MEFs (Fig. 1D). Interestingly, we found that both EZH2 and PRC1 members BMI1 and M33 are also localized at the RD origin of replication in young proliferating MEFs. In contrast, in senescent cells most of the bound EZH2 and M33 protein was lost at all the examined sites along the *INK4a/ARF* locus (Fig. 1D and E). A significant part of BMI1 is still retained at both exon 1b (p19^ARF^) and at the shared exon 2 upon senescence (Fig. 1F) however interestingly, BMI1 completely disappeared from RD (Fig. 1F). Since EZH2 and M33 are lost from the entire locus in senescent cells, these results suggest that BMI1 may bind to some parts of the locus (exon 1b and exon 2) in a manner that is independent of EZH2 and M33, but its binding to RD appears dependent on those two proteins. It has recently been shown that the BMI1 protein was dissociated from the locus at senescence [18]. While we do not explain this difference, the detected BMI1 protein bound at the locus correlates well with the fact that the expression level of Bmi1 is not modified in senescent cells. However, it was demonstrated using genome wide analysis that Polycomb domains can be segregated in two classes: the first occupied by both PRC2 and PRC1 (PRC1-positive) and the second specifically bound by PRC2 (PRC2-only) [23], [24].

**Figure 2:**
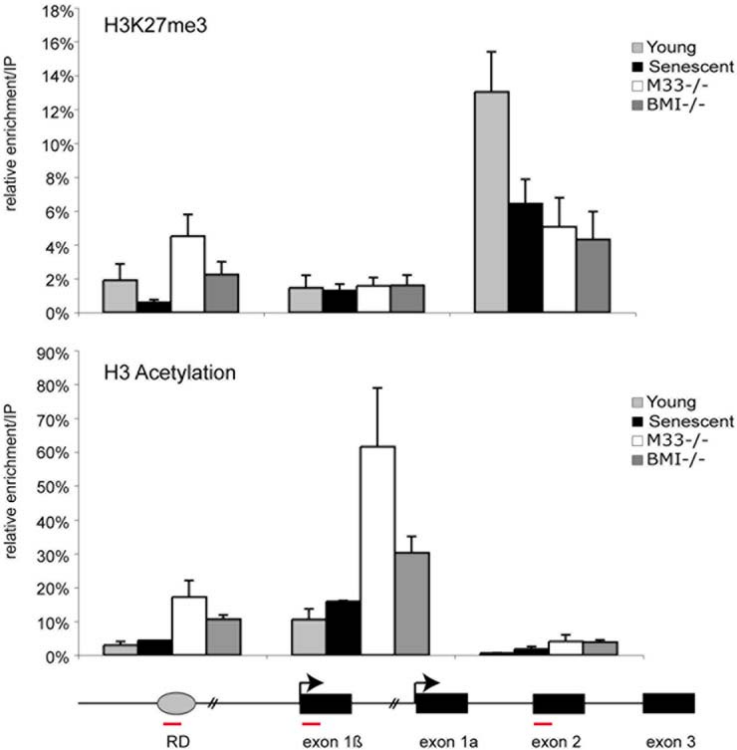
Loss of EZH2 binding and H3K27me3 methylation at the *INK4a/ARF* locus during senescence. P3 proliferating and P10–12 senescent MEFs were subjected to ChIP assays using the indicated antibodies.

Several experiments indicate that H3K27 methylation by E(z) has a critical function in the establishment of transcriptional repression of PRC2 target genes. It has been demonstrated that the PRC2 complex containing E(z)/EZH2 is an active enzyme capable of methylating the histone H3 tail at lysine-9 (K9) and more importantly at K27 [15], [25]. In E(z) Drosophila mutants the loss of functional E(z) induces a loss of Pc-G protein binding to polytene chromosomes [26]. Methylation of histone H3K27 by E(z) protein is strictly required for maintenance of HOX gene silencing in Drosophila [27]. Drosophila Polycomb (Pc or M33 in mouse), a core subunit of PRC1, selectively binds to histone H3 tail peptide trimethylated at K27, suggesting that H3K27me3 may contribute to targeting of PRC1 to HOX genes [28], [29]. We therefore examined the status of methylation of histone H3 at the RD element and at the *INK4a/ARF* locus in young, senescent and Polycomb mutant cells. As shown in figure 2, H3K27me3 marks are lost from the RD element in senescent cells and from the shared exon 2 in senescent, *Bmi1* ^−/−^ and *M33* mutant MEFs. The loss of the H3K27 repressive mark correlates with the higher transcription of *Arf* and *Ink4a* in senescent and Polycomb mutant cells (Fig. 1A). Histone acetylation is generally viewed as a central switch that allows exchange between permissive and repressive chromatin domains in terms of transcriptional competence. It was shown that the levels of p19^Arf^ are strongly upregulated in murine cells treated with histone deacetylase inhibitors (HDACis) [30]. Yet, examination of acetylation of histone H3(K9,K14) in actively cycling and senescent cells shows low level of H3 acetylation at the *INK4a/ARF* locus (Fig. 2). In contrast, we observe a strong enrichment of acetylated H3 at the Arf promoter in *M33* and *Bmi1* knockout MEFs (Fig. 2) similar to the effects observed after treatment with HDACis [30].

### MLL1 is recruited to the *INK4a/ARF* locus during senescence

**Figure 3:**
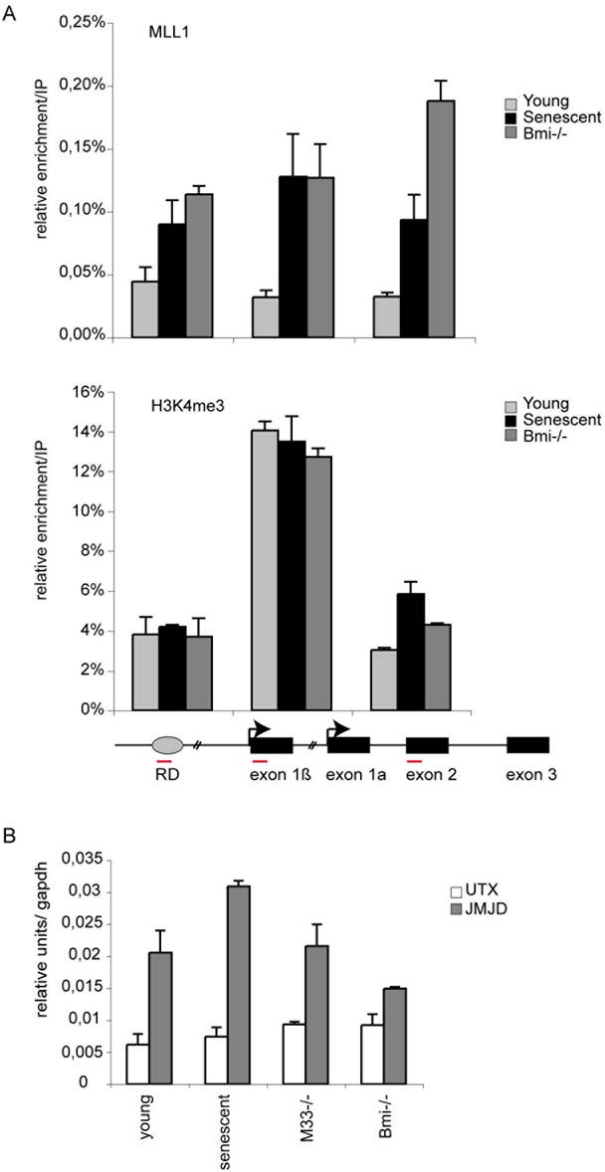
Recruitment of MLL1 at the *INK4a/ARF* locus during senescence. A) ChIP analysis of the RD element, p19^ARF^ exon 1b and p16/19 shared exon 2 using MLL-c antibody and H3K4me3 antibody. B) qPCR analysis of the mRNA levels of UTX and JMJD3 histone demethylase in the indicated cells.

In Drosophila and in mouse the activity of Trx-G/MLL complexes is required to prevent Pc-G-mediated silencing of transcribed Hox genes [31], [32], [33], [34]. MLL1 protein complexes catalyze the trimethylation of H3K4, which is generally associated with active transcription [35]. Accordingly, H3K4me3-modified nucleosomes are specifically enriched at the promoters of active genes [36]. We therefore monitored the binding of the MLL1 protein and the associated H3K4me3 positive transcriptional mark at the RD element and at the *INK4a/ARF* locus in MEFs during senescence and in Polycomb mutant cells. MLL1 was bound to the RD element and to both exon 1b and p16^INK4a^/p19^ARF^ shared exon 2 in young cells (Fig. 3A). However, in both senescent and Polycomb mutant cells we observe a strong enrichment of MLL1 binding at the locus demonstrating that MLL1 participates to the transcription of *Arf* and *Ink4a*. Surprisingly, we did not observe a similar increase of the H3K4 methyl mark during senescence and in mutant cells (Fig. 3A). This positive mark is equally present in young, senescent or Polycomb mutant cells at the *INK4a/ARF* locus. Patterns of methylation at lysine 4 and 27 of histone H3 have been associated with gene activation and repression that are developmentally regulated and are thought to elicit the coordination of lineage specific gene expression programs [37]. Interestingly, in ES cells, the Polycomb Hox target gene promoters often display both H3K4me3 and H3K27me3 marks, and such regions, containing both repressing and activating chromatin modifications, were referred to as “bivalent domains” [38]. In stem cells, these bivalent domains may keep selected genes “poised” for activation. H3K27me3 is a rather stable modification, which could be progressively lost in the absence of PRC2, along with cell divisions [39]. However, studies in ES cells indicated that changes in chromatin associated with Hox gene activation are likely to occur promptly, and involving an appropriate demethylase activity. We therefore monitored the expression of both Jmjd3 and Utx H3K27 histone demethylases. As shown in figure 3B expression of Utx is not modified in young, senescent or *M33* mutant cells. However, transcription of Jmjd3 is significantly induced in senescent MEFs. These results strongly suggest that the upregulation of Jmjd3 (Fig. 3B) and the downregulation of Ezh2 (Fig. 1C) are critical determinants of the transcriptional activation of the *INK4/ARF* locus during senescence. It has been shown that UTX can interact with components of the MLL2 complex [40], [41]. This physical association between enzymes removing the H3K27me3 repressive mark, on the one hand, with protein complexes promoting the deposition of the active H3K4me3 mark, on the other hand, suggests that both activities are required for a rapid and stringent response of target genes. MLL1 is cleaved by Taspase1, generating an N-terminal and a C-terminal fragment, which can heterodimerize *in vitro* [42], [43]. In Drosophila it was demonstrated that TRX-N is present at thousand genomic sites, where no Pc-G binding can be observed. However, it was shown that TRX-C is strongly bound at Pc-G binding sites [44]. These results suggest the C-terminal part of TRX is specifically linked to Pc-G function. It was suggested that Pc-G proteins might repress transcription by anchoring the C-terminal portion of TRX at Polycomb response elements (PREs/TREs) or that constitutive TRX-C binding at PREs/TREs might allow Pc-G target genes to switch their state upon transcriptional induction [44].

### Physical and Functional Association of BMI1 and CDC6 in replication control

**Figure 4:**
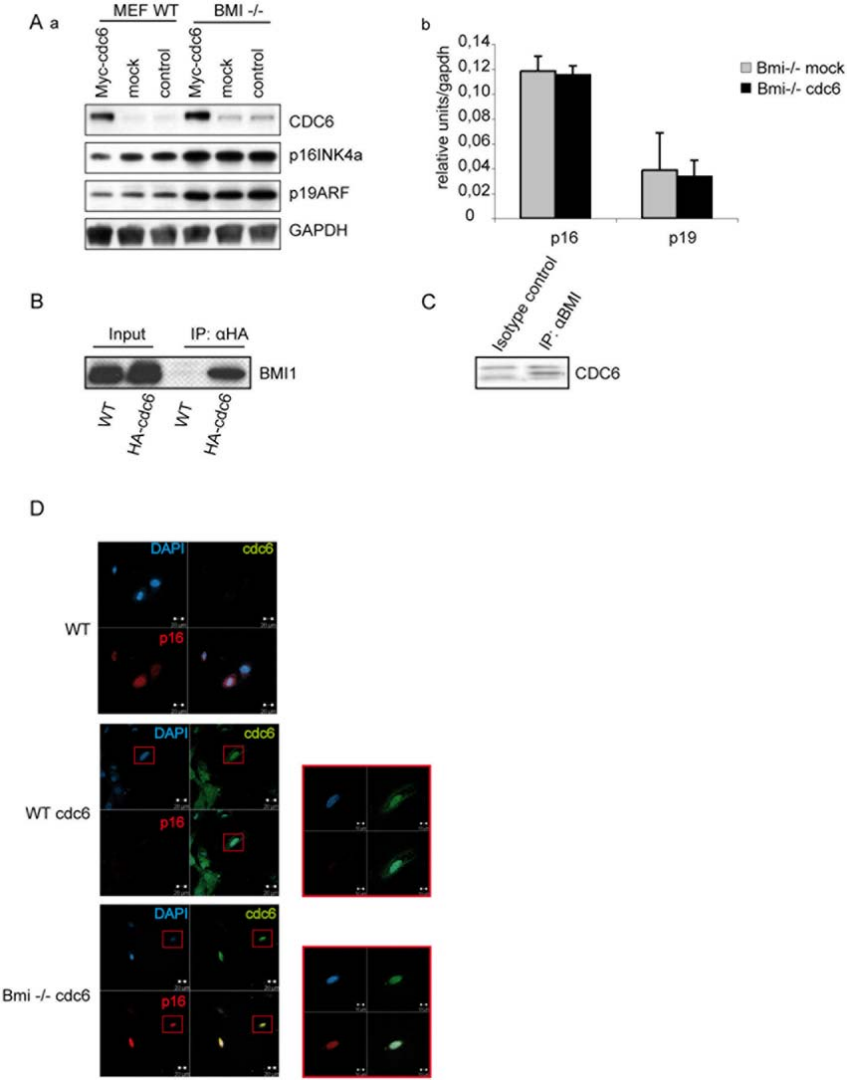
BMI1 interacts with CDC6 and is required for CDC6 repressing function. A) Western Blot analysis of Myc-CDC6 Wild type and *Bmi1* mutant transduced cells. The antibodies used are indicated. GAPDH antibody is used as a loading control. Ab) quantitative PCR experiment showing p16^INK4a^ and p19^ARF^ expression in Myc-CDC6 transduced *Bmi1* mutant cells. B) CDC6 interacts specifically with BMI1: HA immunoprecipitated proteins extracted from HA-CDC6 transfected cells were separated by SDS-PAGE and immunoblotted with a BMI1 antibody. C) anti-Bmi1 immunoprecipitated proteins extracted from wild type thymocytes and immunoblotted with a CDC6 antibody. D) BMI1 is required for INK4a CDC6 mediated repression. Wild type MEFs transfected with Myc-CDC6 were immunostained with a specific antibody against p16^INK4a^ (red) and CDC6 (green) middle panel. Untransfected cells are shown on the upper panel. *Bmi1* mutant cells transfected with Myc-CDC6 (green) express high level of p16 (red) (Bottom panel).

The identification of a DNA replication origin (RD) adjacent to *INK4b* [19], [20] and its high degree of sequence conservation led to ask whether this domain might contribute to the regulation of transcription. Interestingly, overexpressing and loading CDC6 to the RD element, results in the transcriptional repression of all three genes in the *INK4b*–*ARF*–*INK4a* locus [45] leading to increased foci formation and enhanced transformation by oncogenic RAS. Importantly, silencing is accompanied by the recruitment of histone deacetylases and increased methylation of histone H3 on lysine 9 (H3K9), which are hallmarks of heterochromatin. We have demonstrated that both components of the PRC2 and PRC1 complex are localized at the RD element. This prompted us to search for possible physical interactions between the replication complex containing CDC6 and the components of Polycomb complexes. Co-Immunoprecipitation experiments using an antibody against BMI1 demonstrated that CDC6 is associated in a complex with BMI1 (Fig. 4B). Moreover using thymocytes we show that the CDC6-BMI1 interaction occurs in wild type non-transfected cells (Fig. 4C) indicating that the CDC6-BMI1 interaction is not due to the forced expression of CDC6 in transfected MEFs. In order to test if BMI1 is required for Ink4a/Arf repression mediated by CDC6, we transfected CDC6 in *Bmi1* knock out and wild type MEFs. As already described [20], the forced expression of Cdc6 in wild type MEFs decreased the protein levels of ARF and INK4a (Fig. 4A,D). However, overexpression of Cdc6 in mutant *Bmi1* cells failed to mediate Arf or Ink4a repression (Fig. 4A,D) demonstrating that CDC6 induced repression of the *INK4a/ARF* locus is dependent on Polycomb function.

**Figure 5:**
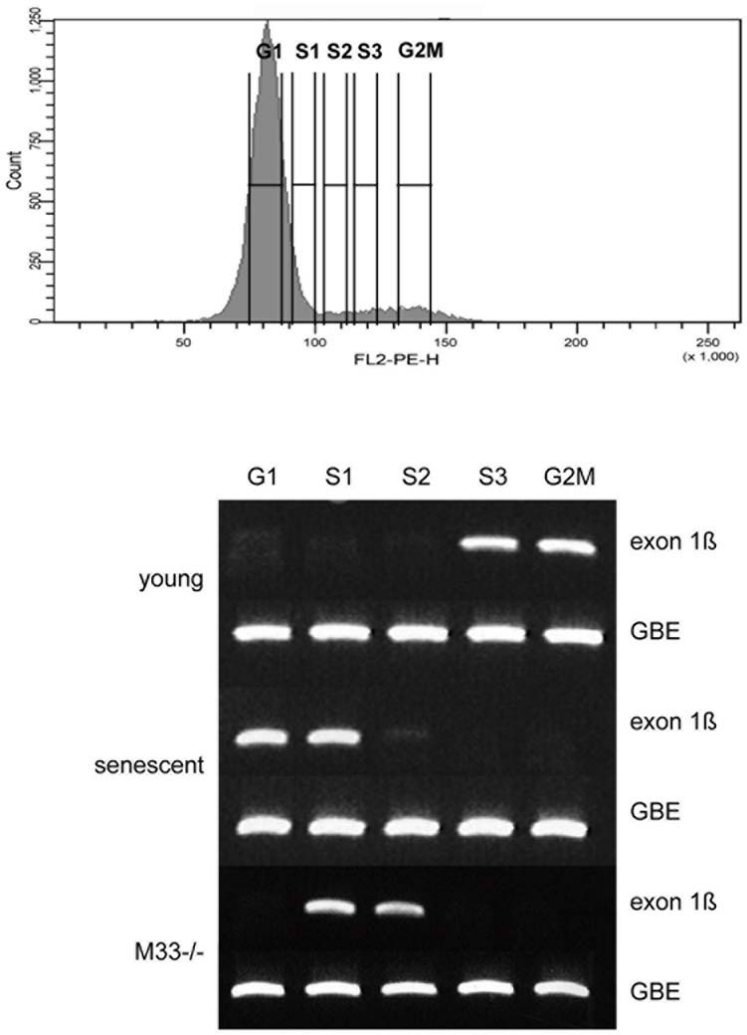
*INK4a/ARF* timing of replication. PCR based analysis of replication timing of the *INK4a/ARF* locus (exon 1b). BrdU pulse labeled cells were stained for DNA content with propidium iodide and sorted by flow cytometry into 5 cell cycle fractions (G1, S1, S2, S3 and G2M) according to DNA content. The Gbe D. melanogaster gene provides a control for recovery of BrdU-labeled DNA.

**Figure 6:**
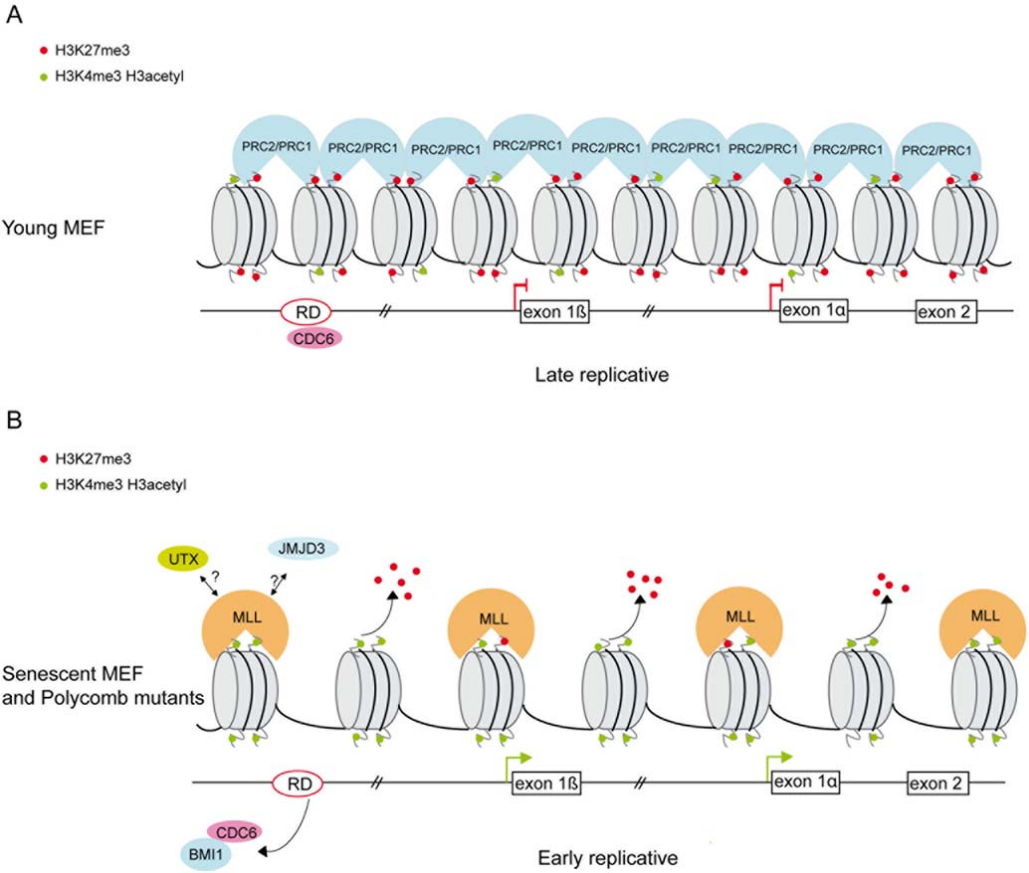
Model for Pc-G and MLL1 proteins in regulation of cellular senescence at the *INK4a/ARF* locus. (A) In young proliferating cells, the PRC2 complex is bound at RD and at the *INK4a/ARF* locus and maintains the levels of H3K27me3. This allows the association of M33 and BMI1-containing PRC1 complex and repression of the INK4a/ARF genes. (B) In senescent or Polycomb mutant cells binding of EZH2 is lost, leading to the disruption of the PRC2 complex, the loss of H3K27me3 and to the recruitment of the MLL1 protein. We propose a model in which Polycomb/MLL1 and JMJD3 epigenetic modifications at the RD element impact the replication timing and the expression of the locus. Moreover, in senescent cells BMI1 binding is specifically lost at the RD element.

Next we asked whether the localization of Polycomb proteins at an origin of replication together with the replication machinery [19] could affect replication of the locus. To investigate whether the induction of senescence could result in a modification of the replication timing of the *INK4a/ARF* locus we examined young proliferating (P3), pre-senescent (P7) and Polycomb *M33* mutant (P3) MEFs. The replication timing was assessed using a PCR based approach [22], [46]. Non-synchronized cells were pulse labeled with 5′Bromodeoxyuridine (BrdU), stained with propidium iodide (PI) and sorted according to DNA content by flow cytometry (Fig. 5). Newly synthesized DNA was isolated by immuno-precipitation with anti-BrdU antibody. As shown in figure 5 the exon 1b (p19^ARF^) is late replicating in young cells which do not express the Arf or Ink4a genes; whereas this region becomes early replicating in pre-senescent and in *M33* mutant cells when the Arf or Ink4a genes are expressed. In higher eukaryotes, it has been observed that the time of replication and transcriptional activity are often correlated; genes which are late replicating are not expressed while transcriptionally active regions are early replicating [47]. In addition, when the transcriptional stage of a gene switches from an active to inactive state, replication timing shifts from early- to late replicating. Importantly, it has recently been demonstrated that histone modifications at an origin of replication serve as a binary switch for controlling the timing of replication of the Beta-globin locus in human [48]. This replication switch is also observed in human diseases such as the fragile X syndrome. FMR1 silencing by the CGG expansion was shown to be mainly attributed to epigenetic regulated transcriptional silencing [49]. The Fmr1 gene normally transcribed is replicated early whereas it becomes silent and late replicating in patients [50], [51]. In yeast it was recently shown that Swi6, an S. pombe counterpart of heterochromatin protein 1 (HP1), is required for early replication of the pericentromeric region and the mat locus [52]. In our study we show that in proliferating MEFs the *INK4a/ARF* locus is silent and late replicating whereas in Polycomb mutant the locus tends to be early replicating and expressed. It has recently been demonstrated in the Encode project that the H3K27me3 mark shows a positive correlation with late replication of large DNA segments [53]. We have demonstrated in senescent and Polycomb mutant cells that the “bivalent” domain at the *INK4a/ARF* locus (H3K27me3 and H3K4me3) is resolved and the locus remains only enriched in H3K4me3 positive marks correlating with the recruitment of MLL1 protein. Jmjd3 overexpression in senescent cells could indicate that this histone demethylase participates in removing the H3K27 marks at the *INK4A/ARF* locus. The epigenetic modifications could be responsible for the observed replication-timing shift at senescence (Fig. 6). Together, our results demonstrate that MLL1 and Polycomb group genes directly control the *INK4a/ARF* locus through chromatin epigenetic modifications and that the loss of the repressive epigenetic marks both in senescent and Polycomb mutant cells at an origin of replication leads to a shift of the replication timing of the locus.
